# Prevalence and Specificity of Anti-HPA and Anti-HLA Antibodies in Patients with Suspected Immune-Mediated Platelet Disorders: A Single-Center Study from Serbia

**DOI:** 10.3390/medicina62040725

**Published:** 2026-04-10

**Authors:** Svetlana Vojvodić, Jasmina Grujić

**Affiliations:** 1 Department of Transfusiology, Faculty of Medicine, University of Novi Sad, 21000 Novi Sad, Serbia; 2 Blood Transfusion Institute of Vojvodina, 21000 Novi Sad, Serbia

**Keywords:** platelet antigens, histocompatibility antigens class I, platelet transfusion, thrombocytopenia, neonatal alloimmune thrombocytopenia

## Abstract

*Background and Objectives:* Alloantibodies directed against human platelet antigens and human leukocyte antigens are implicated in several immune-mediated platelet disorders, including platelet transfusion refractoriness, post-transfusion purpura and fetal and neonatal alloimmune thrombocytopenia. Reliable and simultaneous detection of these antibodies is essential for accurate diagnosis and appropriate clinical management. The aim of this study was to determine the prevalence and specificity spectrum of anti-HLA and anti-HPA alloantibodies in patients with suspected immune-mediated platelet disorders using a multiplex bead-based assay, and to evaluate its diagnostic utility in a Serbian cohort. *Materials and Methods:* A bead-based glycoprotein-specific antibody detection assay was performed using monoclonal antibodies specific for platelet glycoproteins and HLA class I molecules, separately coupled to Luminex microbeads. Serum samples were collected from 259 patients, including 234 patients with thrombocytopenia, 11 with neonatal alloimmune thrombocytopenia, and 14 with suspected platelet transfusion refractoriness. All samples were tested using the PakLx Luminex assay, and results were interpreted with MatchIt! Antibody software. *Results:* Of the 259 tested samples, 72 (27.8%) were positive for HLA and/or platelet-specific antibodies. Among the positive samples, 29.2% contained HLA class I antibodies, 45.8% contained platelet-specific antibodies, and 25% showed combined HLA and platelet antibody positivity. The most frequently detected platelet-specific antibodies were directed against GPIIb/IIIa (HPA-1, -3, -4) and GPIa/IIa (HPA-5). *Conclusions:* This first analysis of platelet alloantibodies in a Serbian cohort demonstrates a high prevalence of antibody positivity in patients with neonatal alloimmune thrombocytopenia and platelet transfusion refractoriness, with anti-HPA-1a as the predominant specificity. The significant association between clinical presentation and antibody profile underscores the need for targeted diagnostic testing. Multiplex bead-based technology provides comprehensive alloantibody detection, facilitating optimized transfusion management in immune-mediated platelet disorders.

## 1. Introduction

Platelets are anucleate cytoplasmic fragments derived from megakaryocytes and play a central role in primary and secondary hemostasis [1,2]. Beyond their well-established involvement in thrombosis and clot formation, platelets are increasingly recognized as active participants in inflammation, innate immunity, tumor biology, regulation of vascular tone, and reproductive processes including implantation and placentation [2,3,4]. Their biological complexity is reflected in a highly specialized membrane structure rich in adhesion receptors and glycoproteins that mediate platelet activation, aggregation, and stabilization of the hemostatic plug [1,5].

The platelet membrane expresses a wide spectrum of antigens that can become targets of alloimmune responses. These include shared antigens, such as human leukocyte antigen (HLA) class I molecules and certain erythrocyte blood group antigens, as well as platelet-specific antigens known as human platelet antigens (HPA) [6,7]. To date, 41 platelet alloantigens have been serologically defined, with six major biallelic systems (HPA-1 to HPA-5 and HPA-15) being of particular clinical relevance [6,8]. These polymorphisms arise from single-nucleotide substitutions in genes encoding platelet membrane glycoproteins, resulting in amino acid substitutions that may trigger alloimmunization when exposed to an antigen-negative individual [6,8].

Alloantibodies directed against HPA and HLAs play a crucial role in several immune-mediated platelet disorders [7,9]. Neonatal alloimmune thrombocytopenia (NAIT) is most frequently associated with anti-HPA-1a antibodies and represents the leading cause of severe thrombocytopenia in otherwise healthy neonates [9,10]. Post-transfusion purpura and platelet transfusion refractoriness are additional clinically significant conditions in which alloantibodies against platelet antigens or HLA molecules contribute to accelerated platelet destruction and therapeutic failure [7,11]. The identification of these antibodies is therefore essential for accurate diagnosis, appropriate transfusion support, and prevention of serious bleeding complications [7,11].

Over time, laboratory methods for the detection of platelet antibodies have evolved from functional assays dependent on platelet activation to antigen-specific techniques capable of identifying the exact glycoprotein target [12]. Third-generation assays, such as Luminex bead-based technology, enable multiplex detection of antibodies while preserving the native conformation of platelet glycoproteins [12,13,14]. This approach allows for simultaneous identification of anti-HPA and anti-HLA antibodies with high sensitivity and specificity, providing a powerful tool for the evaluation of immune-mediated thrombocytopenia [13,14].

Therefore, the present study aimed to determine the prevalence and specificity spectrum of anti-HLA and anti-HPA alloantibodies in patients with suspected immune-mediated platelet disorders, with a particular focus on alloimmune antibody profiles, through the detection and characterization of these antibodies using a multiplex bead-based assay, and to provide the first such analysis in a Serbian cohort.

## 2. Materials and Methods

### 2.1. Study Design and Study Population

This retrospective cross-sectional observational study was conducted at the Blood Transfusion Institute of Vojvodina, using serum samples collected during routine diagnostic procedures. The study included a total of 259 consecutive serum samples collected from patients referred for immunohematological evaluation due to suspected immune-mediated platelet disorders. The study population included 234 patients with thrombocytopenia of various etiologies, 11 patients with clinically suspected or confirmed neonatal alloimmune thrombocytopenia (NAIT), and 14 patients with platelet transfusion refractoriness characterized by inadequate post-transfusion platelet count increments.

Patients with thrombocytopenia were included based on clinical suspicion of immune-mediated etiology, defined as thrombocytopenia of unknown origin after exclusion of non-immune causes or inadequate response to platelet transfusion, where alloimmune platelet destruction could not be excluded. Serum samples were obtained as part of routine diagnostic procedures and stored at −20 °C until analysis. Only the first available serum sample from each patient was included; repeated samples from the same individual were excluded. All laboratory analyses were performed in accordance with institutional standards for immunohematological testing. Due to the retrospective nature of the study, detailed clinical data such as transfusion history, pregnancy history, and comorbidities were not consistently available for all patients. This limitation reflects real-world diagnostic conditions but restricts the ability to perform more detailed risk factor analyses.

### 2.2. Luminex Bead-Based Immunoassay

Detection of anti-human leukocyte antigen (HLA) class I and anti-human platelet antigen (HPA) antibodies was performed using the PakLx Luminex bead-based assay (Immucor, USA), with result interpretation carried out usingMatchIT! Antibody software (version 1.3.0; Immucor, Norcross, GA, USA).

The assay is a qualitative multiplex immunoassay designed to simultaneously detect IgG antibodies directed against HPA-1, HPA-2, HPA-3, HPA-4, HPA-5, glycoprotein IV (GPIV), and HLA class I antigens. The method is based on fluorescently coded magnetic microbeads coated with monoclonal antibodies specific for platelet glycoproteins or HLA class I molecules. These capture antibodies bind detergent-solubilized platelet glycoproteins, preserving conformational epitopes required for antibody recognition.

Patient serum samples were incubated with antigen-coated microbeads, allowing for specific IgG antibodies to bind to their respective targets when present. After washing steps to remove unbound antibodies, bound IgG antibodies were detected using biotinylated anti-human IgG, followed by streptavidin–phycoerythrin conjugate. Fluorescence signals were acquired using a Luminex analyzer, which identifies bead populations based on internal fluorescent signatures and quantifies antibody binding by measuring phycoerythrin emission. Results were expressed as mean fluorescence intensity (MFI). Qualitative interpretation (positive/negative) and cut-off determination were automatically assigned by the MatchIt software according to the manufacturer’s validated algorithm. Borderline or equivocal results were re-tested in accordance with institutional laboratory practice. Screening and identification were performed simultaneously using the PakLx panel, which includes beads coated with HPA-1-5, GPIV, and HLA class I antigens. Samples with MFI values within 10% of the manufacturer’s cut-off were considered borderline and were retested in duplicate; a total of 3 samples fell within this range, and if results remained equivocal, they were classified as negative. Internal quality controls provided by the manufacturer were included in each run. Confirmatory testing by MAIPA was not performed, as the Luminex assay is validated for diagnostic use and has demonstrated high concordance with MAIPA in previous studies [12].

### 2.3. Statistical Analysis

Statistical analysis was performed using standard descriptive and inferential statistical methods. Categorical variables were expressed as absolute numbers and percentages. Differences in the distribution of antibody specificities between patient subgroups were analyzed using the chi-square (χ^2^) test or Fisher’s exact test when appropriate. Odds ratios (ORs) were calculated to assess the association between clinical group (thrombocytopenia, NAIT, and platelet transfusion refractoriness) as the independent variable and antibody positivity as the outcome variable, using thrombocytopenia as the reference group. A *p*-value of less than 0.05 was considered statistically significant. Statistical analyses were performed using SPSS software (version 29.0, IBM Corp., Armonk, NY, USA). Thrombocytopenia patients were chosen as the reference category in odds ratio calculations as they represent the largest and most diagnostically diverse group, allowing for comparison with the clinically more specific NAIT and refractoriness subgroups. No multivariate analysis was performed due to the lack of systematically collected clinical covariates, which limited the ability to adjust for potential confounding factors.

## 3. Results

### 3.1. Study Population

A total of 259 patients were included in the study. The cohort comprised 135 females (52.1%) and 124 males (47.9%). Based on referral indications, 234 patients were evaluated due to thrombocytopenia of various etiologies, 11 patients were investigated for suspected or confirmed neonatal alloimmune thrombocytopenia (NAIT), and 14 patients were assessed due to platelet transfusion refractoriness.

Overall, antibody positivity was detected in 72 patients (27.8%, 95% CI: 22.5–33.6%), whereas 187 patients (72.2%) had no detectable anti-HLA or anti-HPA antibodies.

### 3.2. Association Between Gender and Antibody Positivity

Among female patients, 39 of 135 (28.9%) were antibody positive, while 33 of 124 male patients (26.6%) were antibody positive. Statistical analysis using the chi-square test did not reveal a significant association between gender and antibody positivity (χ^2^ = 0.16, *p* = 0.69), indicating that sensitization was equally distributed between male and female patients (Table 1).

### 3.3. Antibody Positivity According to Clinical Group

A statistically significant association was observed between clinical group and antibody positivity (χ^2^ = 19.7, *p* < 0.001) (Table 2). The prevalence of antibody detection was 23.5% (95% CI: 18.3–29.3%) in patients with thrombocytopenia, 72.7% (95% CI: 43.4–90.3%) in NAIT patients, and 64.3% (95% CI: 38.8–83.7%) in patients with platelet transfusion refractoriness.

Compared to patients with thrombocytopenia, NAIT patients demonstrated an approximately nine-fold increased odds of antibody positivity (OR = 8.7, 95% CI 2.1–36.1), while patients with refractoriness showed nearly six-fold higher odds (OR = 5.9, 95% CI 1.9–18.2).

### 3.4. Distribution of Antibody Types

Among the 72 antibody-positive samples, isolated anti-HPA antibodies were detected in 33 patients (45.83%), isolated anti-HLA class I antibodies were present in 21 patients (29.17%), and combined anti-HLA and anti-HPA antibodies were identified in 18 patients (25%) (Table 3).

In the total cohort, anti-HLA antibodies were detected in 39 patients (15.05%), while anti-HPA antibodies were identified in 51 patients (19.69%).

### 3.5. Distribution of Anti-HPA Specificities

Analysis of individual anti-HPA specificities demonstrated that anti-HPA-1a was the most frequently detected platelet-specific antibody, identified in 30 samples (39.52% of anti-HPA-positive sera). Anti-HPA-5a antibodies were detected in 11 samples (14.47%), followed by anti-HPA-3a antibodies in 10 samples (13.15%). Other detected specificities included anti-HPA-2a, anti-HPA-3b, anti-HPA-4a, and lower frequencies of anti-HPA-1b, anti-HPA-2b, anti-HPA-4b, and anti-HPA-5b (Table 4).

No sensitization against glycoprotein IV (GPIV) was detected in any of the analyzed samples.

## 4. Discussion

The present study demonstrates a statistically significant association between clinical indication and the presence of anti-HLA and anti-HPA antibodies, while no association was observed between gender and the presence of antibodies. The overall antibody prevalence of 27.79% confirms that alloimmunization remains a clinically relevant issue in patients referred for evaluation of immune-mediated platelet disorders. This prevalence is comparable to previously reported rates in transfusion-exposed populations, particularly in patients with suspected platelet refractoriness, where rates between 20% and 50% have been described depending on the number of transfusions and underlying diagnosis [13,14]. However, direct comparisons between studies are challenging due to differences in patient selection, assay sensitivity, and definitions of positivity. Nevertheless, our findings establish a baseline for the Serbian population, for which no such data have previously been available.

A highly significant association was observed between the clinical group and antibody detection. Patients with NAIT demonstrated the highest prevalence of antibody positivity (72.7%), followed by patients with platelet transfusion refractoriness (64.3%). These findings are consistent with the established immunopathogenesis of NAIT, where maternal alloimmunization against fetal platelet antigens, most commonly HPA-1a, leads to severe neonatal thrombocytopenia [15,16]. The 72.7% detection rate in our NAIT subgroup is somewhat lower than the 85–95% typically reported in large NAIT cohorts [15,17]. This discrepancy may be explained by the small sample size (*n* = 11), potential inclusion of cases where blood samples were drawn after platelet transfusions, or the presence of antibodies against low-frequency HPA systems not covered by the PakLx panel. Additionally, some clinically suspected NAIT cases may have non-immune etiologies, including congenital thrombocytopenia or perinatal asphyxia, which would yield negative antibody results [16].

Anti-HPA-1a antibodies account for approximately 75–80% of NAIT cases in Caucasian populations, with anti-HPA-5b and other specificities occurring less frequently [15,17]. In our study, anti-HPA-1a was also the most common specificity, accounting for 39.5% of HPA-positive sera. Although this proportion is lower than that reported in NAIT-specific cohorts, this difference is expected given the heterogeneity of our study population, which included patients with thrombocytopenia of various etiologies in addition to NAIT. When analyzed within the NAIT subgroup, anti-HPA-1a was detected in 75% of antibody-positive cases, consistent with the literature [15,16]. These findings are also in line with studies using multiplex bead-based assays in mixed patient populations, where anti-HPA-1a consistently emerges as the predominant specificity, followed by anti-HPA-5b and anti-HPA-3a [10,13].

Similarly, platelet transfusion refractoriness is frequently associated with alloimmunization, particularly against HLA class I antigens (reported in 50–80% of cases) and, to a lesser extent, platelet-specific antigens (10–20%) [18,19]. In our refractoriness subgroup, isolated anti-HLA antibodies, isolated anti-HPA antibodies, and combined sensitization were each observed in approximately one-third of antibody-positive cases. The relatively high proportion of isolated anti-HPA antibodies in refractoriness patients may reflect the inclusion of patients with immune thrombocytopenia who received transfusions, or it may suggest that platelet-specific antibodies are underrecognized contributors to poor transfusion outcomes. Repeated transfusion exposure significantly increases the likelihood of antibody formation, especially in chronically transfused patients with hematologic malignancies or bone marrow failure syndromes. The nearly six-fold increased odds of antibody positivity observed in our refractoriness subgroup align with previously published data emphasizing the immunologic component of poor platelet increment following transfusion [17,18,19].

The absence of a significant association between gender and antibody positivity suggests that in a diagnostically selected cohort, antibody positivity is influenced more by antigen exposure than by sex-related factors. While pregnancy is a well-recognized risk factor for alloimmunization in women, transfusion-related exposure contributes substantially to antibody formation in both sexes [20]. In our cohort, obstetric history was not systematically collected, which represents a limitation when interpreting potential sex-related differences in alloimmunization. Nevertheless, the comparable rates between males (26.6%) and females (28.9%) suggest that pregnancy alone may not fully explain the overall antibody prevalence. Other sources of antigen exposure, such as previous transfusions, which were not systematically recorded in this study, may have contributed to sensitization in both sexes. Future studies should collect detailed transfusion and obstetric histories to clarify the relative contributions of each.

Isolated anti-HPA antibodies were detected more frequently than isolated anti-HLA antibodies, and combined antibody positivity was identified in 25% of antibody-positive cases. The presence of both anti-HLA and anti-HPA antibodies may complicate transfusion management and may necessitate both HLA-matched and antigen-compatible platelet support [17,21]. In such patients, random donor platelets are unlikely to achieve adequate post-transfusion increments, and specialized donor selection, either through HLA-typed plateletpheresis donors or crossmatching, becomes essential. Multiplex detection methods are therefore particularly valuable in complex sensitization patterns, as they provide a complete antibody profile from a single sample.

Anti-HPA-1a was the predominant platelet-specific antibody identified in this study, accounting for nearly 40% of anti-HPA-positive sera. The strong immunogenicity of HPA-1a is well established and has been linked to several biological and genetic factors. The HPA-1a epitope is located on glycoprotein IIIa (integrin β3), part of the highly abundant GPIIb/IIIa complex, which is present at approximately 80,000 copies per platelet [22]. Additionally, the immune response to HPA-1a is T-cell dependent and strongly associated with the HLA-DRB3*01:01 allele, present in more than 90% of immunized women of European descent [21]. This genetic restriction significantly increases the likelihood of alloimmunization following antigen exposure.

In line with our findings, the predominance of antibodies targeting glycoprotein IIb/IIIa-associated HPAs (HPA-1, -3, -4) and GPIa/IIa-associated HPA-5 is consistent with previous reports indicating that higher molecular weight glycoproteins with abundant surface expression demonstrate increased immunogenic potential [15]. Glycoprotein IIb/IIIa is a highly abundant platelet surface receptor, which may explain why antibodies against its associated polymorphisms (HPA-1, HPA-3, HPA-4) are frequently observed in alloimmunized patients [10,13]. In contrast, no antibodies against GPIV (CD36) were detected in our cohort. This finding is consistent with reports that anti-CD36 antibodies are relatively uncommon in Caucasian populations, whereas they are more frequently described in Asian and African populations due to genetic polymorphisms in the CD36 gene [23]. This highlights the importance of population-specific data in platelet immunology.

The Luminex bead-based immunoassay used in this study enabled simultaneous detection of anti-HLA and anti-HPA antibodies with high analytical sensitivity. Compared with earlier generation assays such as monoclonal antibody immobilization of platelet antigens (MAIPA), Luminex technology allows for multiplex detection, requires smaller serum volumes, and provides objective fluorescence-based interpretation [7,14]. Previous comparative studies have demonstrated comparable or superior sensitivity of Luminex platforms in detecting anti-HPA antibodies, particularly in low-titer sera [10]. A recent single-laboratory comparison of MAIPA, platelet immunofluorescence testing (PIFT), and the PakLx Luminex assay reported high concordance (94–97%) between Luminex and MAIPA for anti-HPA-1a detection, with Luminex showing slightly higher sensitivity for low-level antibodies [12]. This is particularly relevant for NAIT, where maternal antibody titers may decline rapidly after delivery, and for patients with weak sensitization who may still experience clinical sequelae [12].

Our findings have several practical implications for transfusion medicine in Serbia and the region. The high prevalence of anti-HPA-1a antibodies supports the consideration of antenatal screening for HPA-1a incompatibility in pregnant women, as has been evaluated in several European countries (e.g., the Netherlands) [24]. Such screening could identify at-risk pregnancies and enable timely intervention, including antenatal intravenous immunoglobulin or early delivery [4,9,14]. In addition, for patients with platelet transfusion refractoriness, our data suggest that both HLA and HPA antibody testing should be performed routinely, as isolated anti-HPA antibodies were present in a substantial proportion of cases. Relying solely on HLA-matched platelets may be insufficient in such patients, as HPA alloantibodies are recognized contributors to poor transfusion outcomes [11,16]. Finally, the establishment of a registry of HPA-typed platelet donors in Serbia would facilitate the provision of antigen-negative platelets for alloimmunized patients, particularly those with anti-HPA-1a.

This study has several strengths. First, the use of a multiplex Luminex platform enabled simultaneous detection of both anti-HPA and anti-HLA antibodies in a real-world diagnostic setting. Second, the inclusion of clinically well-defined patient subgroups (thrombocytopenia, NAIT, and platelet transfusion refractoriness) enabled meaningful comparison of antibody prevalence across different immune-mediated platelet disorders. Third, the relatively large overall sample size (*n* = 259) provides reasonable precision for the prevalence estimates. Although the general mechanisms of platelet alloimmunization are well established, region-specific data on the distribution and specificity of anti-HPA and anti-HLA antibodies remain limited. In this context, to the best of our knowledge, this is the first study to report the distribution of HPA and HLA alloantibodies in a Serbian cohort, providing clinically relevant baseline data for future research and transfusion practice in the region. However, certain limitations should be acknowledged. The small sample sizes in the NAIT (*n* = 11) and platelet transfusion refractoriness (*n* = 14) subgroups limit statistical power and result in wide confidence intervals around the odds ratio estimates (Table 2); therefore, these findings should be interpreted with caution. Relevant clinical variables such as transfusion history and pregnancy history were not systematically collected, precluding the use of multivariate analysis to adjust for potential confounders. Future studies incorporating these variables would allow for a more robust assessment of factors associated with alloimmunization. Additionally, HPA genotyping was not performed, which could have provided further insight into antigen–antibody compatibility and immunization risk. Furthermore, confirmatory testing using the MAIPA assay was not performed. Although the PakLx Luminex assay has demonstrated high concordance with MAIPA in previous studies, the absence of independent confirmatory testing may limit the precise characterization of specific anti-HPA antibodies and should be considered a methodological limitation [12].

Future studies should aim to include prospective multicenter enrollment across Serbia to increase sample sizes, particularly for NAIT and refractoriness subgroups. Comparison of multiplex Luminex assays with established methods such as PIFT and MAIPA would allow for more precise evaluation of assay performance. Integration of molecular typing for both HPA and HLA would enable genotype-guided donor selection and precise determination of immunization risk. Correlation of antibody profiles with clinical outcomes, including bleeding scores, intracranial hemorrhage in neonates, and corrected count increments following transfusion, would strengthen the clinical relevance of laboratory findings and improve risk stratification and transfusion outcomes. Finally, a cost-effectiveness analysis of routine Luminex-based screening versus targeted testing based on clinical suspicion would help guide resource allocation in the Serbian healthcare system.

## 5. Conclusions

In conclusion, our findings confirm a high prevalence of alloantibodies in patients with NAIT and platelet transfusion refractoriness and identify anti-HPA-1a as the predominant platelet-specific antibody. The observed association between clinical indication and antibody positivity underscores the importance of targeted immunohematological testing in patients with suspected immune-mediated thrombocytopenia.

In addition to confirming established immunohematological patterns, this study provides real-world, region-specific data that may support the optimization of diagnostic strategies and transfusion management. Multiplex Luminex bead-based assays represent a valuable tool for comprehensive alloantibody detection in routine clinical practice, enabling more precise and efficient clinical decision-making.

Future prospective studies with larger cohorts, particularly in NAIT and platelet transfusion refractoriness, are warranted to validate these findings and to further evaluate their impact on clinical outcomes and transfusion strategies.

## Figures and Tables

**Table 1:** Distribution of antibody positivity by gender.

Gender	Positive *n* (%)	Negative *n* (%)
Female	39 (28.9)	96 (71.1)
Male	33 (26.6)	91 (73.4)

χ^2^ = 0.16, *p* = 0.69.

**Table 2:** Distribution of antibody positivity across clinical groups.

Clinical Group	Positive *n* (%)	95% CI	Negative *n* (%)	OR (95% CI)
Thrombocytopenia	55 (23.5)	18.3–29.3	179 (76.5)	1.0 (Reference)
NAIT	8 (72.7)	43.4–90.3	3 (27.3)	8.7 (2.1–36.1)
Refractoriness	9 (64.3)	38.8–83.7	5 (35.7)	5.9 (1.9–18.2)

Overall χ^2^ = 19.7, *p* < 0.001.

**Table 3:** Frequency of antibody types in the study cohort.

Antibody Status	*n* (% of Total)
Anti-HLA only	21 (8.1)
Anti-HPA only	33 (12.7)
Anti-HLA + HPA	18 (6.9)
Negative	187 (72.2)

**Table 4:** Frequency of individual anti-HPA specificities.

Antibody	*n*	% of HPA-Positive
HPA-1a	30	39.52
HPA-5a	11	14.47
HPA-3a	10	13.15
HPA-2a	6	7.89
HPA-5b	6	7.89
HPA-3b	5	6.57
HPA-4a	4	5.26
HPA-1b	2	2.63
HPA-2b	1	1.31
HPA-4b	1	1.31

## Data Availability

The data presented in this study are available upon request from the corresponding author. The data are not publicly available due to privacy and ethical considerations.

## Abbreviations

The following abbreviations are used in this manuscript:

HPA	Human Platelet Antigen
HLA	Human Leukocyte Antigen
NAIT	Neonatal Alloimmune Thrombocytopenia
MFI	Mean Fluorescence Intensity
GP	Glycoprotein
GPIV	Glycoprotein IV
IgG	Immunoglobulin G
MAIPA	Monoclonal Antibody Immobilization of Platelet Antigens
OR	Odds Ratio
CI	Confidence Interval
χ^2^	Chi-square test
SPSS	Statistical Package for the Social Sciences
PakLx	Platelet Antibody Luminex Assay
PIFT	Platelet Immunofluorescence Testing
